# Cost-effectiveness of CT perfusion for the detection of large vessel occlusion acute ischemic stroke followed by endovascular treatment: a model-based health economic evaluation study

**DOI:** 10.1007/s00330-023-10119-y

**Published:** 2023-09-20

**Authors:** Henk van Voorst, Jan W. Hoving, Miou S. Koopman, Jasper D. Daems, Daan Peerlings, Erik Buskens, Hester F. Lingsma, Ludo F. M. Beenen, Hugo W. A. M. de Jong, Olvert A. Berkhemer, Wim H. van Zwam, Yvo B. W. E. M. Roos, Marianne A. A. van Walderveen, Ido van den Wijngaard, Diederik W. J. Dippel, Albert J. Yoo, Bruce C. V. Campbell, Wolfgang G. Kunz, Bart J. Emmer, Charles B. L. M. Majoie, for the CLEOPATRA investigators, for the CONTRAST consortium, Rick van Nuland, Aad van der Lugt, Adriaan van Es, Pieter-Jan van Doormaal, René van den Berg, Ludo F. M. Beenen, Stefan D. Roosendaal, Alida Annechien Postma, Lonneke S. F. Yo, G. Menno Krietemeijer, Geert J. Lycklama à Nijeholt, Jasper M. Martens, Sebastiaan Hammer, Anton Meijer, Reinoud P. H. Bokkers, Anouk van der Hoorn, Dick Gerrits, Robert van Oostenbrugge, Jonathan M. Coutinho, Martine Truijman, Julie Staals, H. Bart van der Worp, J. Boogaarts, Ben Jansen, Sanne Zinkstok, Martine Truijman, Julie Staals, Peter Koudstaal, Koos Keizer, Sanne Manschot, Jelis Boiten, Henk Kerkhoff, Vicky Chalos, Adriaan Versteeg, Lennard Wolff, Matthijs van der Sluijs, Manon L. Tolhuisen, Hugo ten Cate, Moniek de Maat, Samantha Donse-Donkel, Heleen van Beusekom, Aladdin Taha, Aarazo Barakzie, Rob van de Graaf, Wouter van der Steen, Kilian M. Treurniet, Sophie van den Berg, Natalie LeCouffe, Manon Kappelhof, Rik Reinink, Leon Rinkel, Josje Brouwer, Agnetha Bruggeman, Robert-Jan Goldhoorn, Wouter Hinsenveld, Anne Pirson, Susan Olthuis, Simone Uniken Venema, Sjan Teeselink, Lotte Sondag, Sabine Collette, Martin Sterrenberg, Naziha el Ghannouti, Laurine van der Steen, Sabrina Verheesen, Jeannique Vranken, Ayla van Ahee, Hester Bongenaar, Maylee Smallegange, Lida Tilet, Joke de Meris, Michelle Simons, Wilma Pellikaan, Wilma van Wijngaarden, Kitty Blauwendraat, Yvonne Drabbe, Michelle Sandiman-Lefeber, Anke Katthöfer, Eva Ponjee, Rieke Eilander, Anja van Loon, Karin Kraus, Suze Kooij, Annemarie Slotboom, Friedus van der Minne, Esther Santegoets, Leontien Heiligers, Yvonne Martens, for the MR CLEAN Registry Investigators, Jan Albert Vos, Ivo G. H. Jansen, Maxim J. H. L. Mulder, Kars C. J. Compagne, Sanne J. den Hartog, Bob Roozenbeek, Wouter J. Schonewille, Marieke J. H. Wermer, Jeannette Hofmeijer, Geert J. Lycklama à Nijeholt, Sebastiaan F. de Bruijn, Lukas C. van Dijk, Rob H. Lo, Ewoud J. van Dijk, Hieronymus D. Boogaarts, J. de Vries, Paul L. M. de Kort, Julia van Tuijl, Jo P. Peluso, Puck Fransen, Jan S. P. van den Berg, Boudewijn A. A. M. van Hasselt, Leo A. M. Aerden, René J. Dallinga, Maarten Uyttenboogaart, Omid Eschgi, Tobien H. C. M. L. Schreuder, Roel J. J. Heijboer, Koos Keizer, Heleen M. den Hertog, Emiel J. C. Sturm, Marieke E. S. Sprengers, Sjoerd F. M. Jenniskens, Bas F. W. van der Kallen, Joost Bot, Elyas Ghariq, Marc P. van Proosdij, Wouter Dinkelaar, Auke P. A. Appelman, Bas Hammer, Sjoert Pegge, Saman Vinke, H. Zwenneke Flach, Rita Sprengers, Marjan Elfrink, Marjolein Vossers, Joke de Meris, Tamara Vermeulen, Annet Geerlings, Gina van Vemde, Tiny Simons, Gert Messchendorp, Nynke Nicolaij, Karin Bodde, Sandra Kleijn, Jasmijn Lodico, Hanneke Droste, Maureen Wollaert, D. Jeurrissen, Erna Bos, Michelle Sandiman, Nicoline Aaldering, Berber Zweedijk, Jocova Vervoort, Sharon Romviel, Karin Kanselaar, Denn Barning, Esmee Venema, Ralph R. Geuskens, Tim van Straaten, Saliha Ergezen, Roger R. M. Harmsma, Daan Muijres, Anouk de Jong, Anna M. M. Boers, P. F. C. Groot, Marieke A. Mens, Katinka R. van Kranendonk, Heitor Alves, Annick J. Weterings, Eleonora L. F. Kirkels, Eva J. H. F. Voogd, Lieve M. Schupp, Adrien E. D. Groot, Praneeta R. Konduri, Haryadi Prasetya, Nerea Arrarte-Terreros, Lucas A. Ramos

**Affiliations:** 1 Department of Radiology and Nuclear Medicine, Amsterdam UMC Location University of Amsterdam, Meibergdreef 9, 1105 AZ Amsterdam, the Netherlands; 2 Department of Biomedical Engineering and Physics, Amsterdam UMC Location University of Amsterdam, Amsterdam, the Netherlands; 3 Department of Public Health, Erasmus University Medical Center, Rotterdam, the Netherlands; 4 Department of Neurology, Erasmus University Medical Center, Rotterdam, the Netherlands; 5 Department of Radiology, University Medical Center Utrecht, Utrecht, the Netherlands; 6 Department of Epidemiology, University Medical Center Groningen, University of Groningen, Groningen, the Netherlands; 7 Department of Radiology and Nuclear Medicine, Erasmus University Medical Center, Rotterdam, the Netherlands; 8 Department of Radiology and Nuclear Medicine, Cardiovascular Research Institute Maastricht (CARIM), Maastricht University Medical Center+, Maastricht, the Netherlands; 9 Department of Neurology, Amsterdam UMC Location University of Amsterdam, Amsterdam, the Netherlands; 10 Department of Radiology, Leiden University Medical Center, Leiden, the Netherlands; 11 Department of Neurology, Haaglanden Medical Center, The Hague, the Netherlands; 12 Department of Radiology, Texas Stroke Institute, Dallas-Fort Worth, TX USA; 13 Department of Medicine and Neurology, Melbourne Brain Center, Royal Melbourne Hospital, University of Melbourne, Melbourne, Australia; 14 Department of Radiology, University Hospital, LMU Munich, Munich, Germany

**Keywords:** Ischemic stroke, Four-dimensional computed tomography, Diagnosis, Thrombectomy, Health Care Economics and Organizations

## Abstract

**Objectives:**

CT perfusion (CTP) has been suggested to increase the rate of large vessel occlusion (LVO) detection in patients suspected of acute ischemic stroke (AIS) if used in addition to a standard diagnostic imaging regime of CT angiography (CTA) and non-contrast CT (NCCT). The aim of this study was to estimate the costs and health effects of additional CTP for endovascular treatment (EVT)–eligible occlusion detection using model-based analyses.

**Methods:**

In this Dutch, nationwide retrospective cohort study with model-based health economic evaluation, data from 701 EVT-treated patients with available CTP results were included (January 2018–March 2022; trialregister.nl:NL7974). We compared a cohort undergoing NCCT, CTA, and CTP (NCCT + CTA + CTP) with a generated counterfactual where NCCT and CTA (NCCT + CTA) was used for LVO detection. The NCCT + CTA strategy was simulated using diagnostic accuracy values and EVT effects from the literature. A Markov model was used to simulate 10-year follow-up. We adopted a healthcare payer perspective for costs in euros and health gains in quality-adjusted life years (QALYs). The primary outcome was the net monetary benefit (NMB) at a willingness to pay of €80,000; secondary outcomes were the difference between LVO detection strategies in QALYs (ΔQALY) and costs (ΔCosts) per LVO patient.

**Results:**

We included 701 patients (median age: 72, IQR: [62–81]) years). Per LVO patient, CTP-based occlusion detection resulted in cost savings (ΔCosts median: € − 2671, IQR: [€ − 4721; € − 731]), a health gain (ΔQALY median: 0.073, IQR: [0.044; 0.104]), and a positive NMB (median: €8436, IQR: [5565; 11,876]) per LVO patient.

**Conclusion:**

CTP-based screening of suspected stroke patients for an endovascular treatment eligible large vessel occlusion was cost-effective.

**Clinical relevance statement.:**

Although CTP-based patient selection for endovascular treatment has been recently suggested to result in worse patient outcomes after ischemic stroke, an alternative CTP-based screening for endovascular treatable occlusions is cost-effective.

**Key Points:**

*• Using CT perfusion to detect an endovascular treatment-eligible occlusions resulted in a health gain and cost savings during 10 years of follow-up.*

*• Depending on the screening costs related to the number of patients needed to image with CT perfusion, cost savings could be considerable (median: € − 3857, IQR: [€ − 5907; € − 1916] per patient).*

*• As the gain in quality adjusted life years was most affected by the sensitivity of CT perfusion-based occlusion detection, additional studies for the diagnostic accuracy of CT perfusion for occlusion detection are required.*

**Supplementary information:**

The online version contains supplementary material available at 10.1007/s00330-023-10119-y.

## Introduction

Brain tissue perfusion maps derived from computed tomography perfusion (CTP) have been suggested to improve occlusion detection in acute ischemic stroke (AIS) patients if used in addition to CT angiography (CTA) and non-contrast CT (NCCT) [1–4]. Although CTP is primarily considered to select patients for endovascular treatment (EVT) [5], screening all suspected AIS patients presenting within 6 h after symptom onset with CTP could also enhance the detection of patients with a large vessel occlusion (LVO) EVT, resulting in more patients who benefit from EVT and less missed occlusions [6]. Although EVT compared to best medical management is considered cost-effective [7], it remains unclear to what extent the direct costs of screening a large group of patients with CTP for EVT-eligible occlusions result in long-term health gains and cost savings.

Several studies found that adding CTP to an imaging regime of non-contrast-enhanced CT (NCCT) and CT angiography (CTA) enhances the sensitivity for arterial occlusion detection [1–4]. Moreover, the sensitivity gain of adding CTP was between 0 and 20% [1–4] — depending on the experience of the neuroradiologist and the occlusion location. Since EVT has vastly improved outcomes of AIS patients with a large vessel occlusion (LVO) [6, 8], the total quality-adjusted life-years (QALYs) of patients after an AIS can be increased by providing EVT to all eligible patients.

Two previous health economic evaluations concluded that CTP was cost-effective when used jointly for EVT- and intravenous thrombolysis–eligible occlusion detection and to exclude patients with severe ischemia for whom EVT may potentially be harmful [9, 10]. However, these studies considered deterministic fixed estimates for the value of additional CTP for EVT-eligible LVO detection that do not correspond with recent findings [1, 2]. In addition, the benefit of CTP-based occlusion detection may be higher for less-experienced physicians compared to experienced neuroradiologists. Furthermore, variations in the proportion of patients with an EVT-eligible occlusion compared to the overall population presenting with AIS symptoms at the emergency department alter the number of patients needed to image (NNI) to detect an LVO and increase the total costs of CTP. Finally, the two studies considered a US perspective that might not apply to other healthcare systems [9, 10].

In this study, we aimed to estimate the long-term costs and health effects of adding CTP (NCCT + CTA + CTP) for LVO detection to a standard imaging protocol of NCCT and CTA (NCCT + CTA) in Dutch patients suspected of AIS presenting within 6 h after symptom onset at an EVT-capable hospital. Furthermore, we aimed to analyze the effect of variations in the sensitivity of NCCT + CTA + CTP compared to NCCT + CTA-based LVO detection, the number of CTPs needed to acquire (NNI) before an LVO was detected, and the benefit of EVT.

## Methods

### Study design

In this study, a cohort of patients that received EVT after NCCT + CTA + CTP-based LVO detection with 90-day functional outcome according to the modified Rankin Scale (mRS) was used to simulate long-term mRS and a cohort with 90-day mRS after NCCT + CTA-based LVO detection. An EVT-eligible LVO was defined as occlusion of the internal carotid artery-(terminus) (ICA/ICA-T), the M1 or proximal M2 segment of the middle cerebral artery. To generate the NCCT + CTA cohort, a percentage of patients was simulated as if they did not receive EVT due to a missed LVO. This percentage of missed LVOs was based on the difference in sensitivity between NCCT + CTA + CTP- and NCCT + CTA-based LVO detection found in a literature search (Online Supplement A); this was referred to as the sensitivity difference [1–4]. We did not consider the difference in specificity or positive predictive value due to additional CTP because the relative difference between the imaging strategies would be small and the negative health effects and additional costs of a futile transfer to the angio suite are assumed to be negligible on a population basis. For patients that would not have received EVT under the NCCT + CTA regime, the observed mRS in the included cohort was reduced using available ORs for EVT effect from the literature (Online Supplement B) [6, 11]. To include the additional costs of CTP in the NCCT + CTA + CTP arm, the NNI was used to add additional screening costs per patient with an LVO; for each detected LVO, there would be numerous patients without an LVO that were screened with CTP and related costs (Online Supplement C).

### Patient level data

We included patients from the Cost-effectiveness of CT perfusion for Patients with Acute Ischemic Stroke (CLEOPATRA) healthcare evaluation study [12] in the Netherlands that recruited patients originally included in the MR CLEAN NO IV [13], MR CLEAN MED [14], and MR CLEAN Registry [15] (January 2018–March 2022; trialregister.nl:NL7974). We only included data from EVT-treated patients with available CTP imaging who presented within 6 h after stroke symptom onset in an EVT capable hospital. Minor protocol deviations and CTP processing methods are available in Online Supplement D and E. This study was conducted according to the Helsinki agreement; part of the data has previously been reported (Online Supplement F).

### Modeling approach

**Fig. 1:**
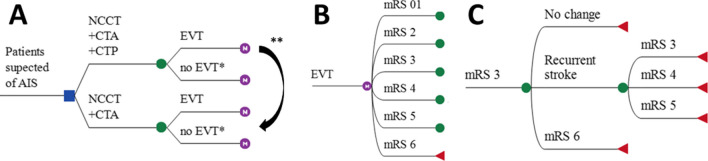
Markov model structure. **A** Patients presenting within 6 h after stroke symptom onset at an endovascular treatment (EVT)–capable stroke center are subject to one of the following diagnostic imaging protocols for EVT-eligible occlusion detection: (1) non-contrast CT (NCCT), CT angiography (CTA), and CT perfusion (CTP), or (2) NCCT and CTA. No EVT*: In the NCCT + CTA + CTP arm, the number of patients without an EVT-eligible occlusion (no EVT) was computed using the number needed to image (NNI) calculations. Costs of CTP-based screening of non-EVT-eligible occlusions were multiplied with the NNI and added to the overall costs of this simulated arm, in the models’ CTP—no EVT group did not suffer any health consequences and was not further simulated. In the NCCT + CTA arm, the no EVT compromised all patients from the NCCT + CTA + CTP arm in addition to all patients that were missed due to less optimal EVT-eligible occlusion screening. The long-term modified Rankin Scale (mRS) of the missed EVT-eligible occlusion group was further simulated. ** The sensitivity gain due to CTP-based EVT-eligible occlusion detection was used to compute the size of the group of missed EVT-eligible occlusions if a diagnostic imaging protocol consisting of NCCT + CTA was used. **B** The 90-day mRS was modeled after EVT or no EVT. **C** Yearly mRS transitions were modeled based on death and recurrent stroke rates beyond 90 days after stroke. EVT, endovascular treatment; NCCT, non-contrast enhanced CT; CTA, CT angiography; mRS, modified Rankin scale

We simulated 5- and 10-year follow-up using a Markov model with patient-level microsimulations. The Markov model was previously described and consisted of a short-term 90-day post-AIS model followed by a long-term yearly model to simulate functional outcome using the modified Rankin Scale (mRS) (Fig. 1) [12, 16]. In the short-term model, we simulated the 90-day mRS of patients that received EVT and those who did not based on NCCT + CTA + CTP- or NCCT + CTA-based LVO detection. In the long-term model, we simulated yearly mRS deterioration after 90 days based on the probability of stroke recurrence [17] and death [18] inflated with patient-specific hazard ratios (HR) [19]. Python scripts for the simulations are made publicly available (github.com/henkvanvoorst92/CLEOPATRA).

### Costs and QALYs

We used mRS over the simulated period to compute cumulative costs from a healthcare payer perspective and QALYs [20]. The methodology for acute care and mRS related follow-up QALYs and costs has previously been described [12, 20]. QALYs were computed per mRS sub-score per year based on 391 patients with 2-year follow-up and available EuroQoL 5D questionnaires [8]. Follow-up costs for the first, second, and third year onward per mRS sub-score included the following: acute setting treatment cost, in-hospital costs, outpatient clinic visits, rehabilitation, formal homecare, and long-term institutionalized costs [8]. Acute care costs included NCCT, CTA, CTP, EVT, and IVT if applicable based on reference prices from the institute of Medical Technological Assessment, Rotterdam, the Netherlands [12, 20]. Acute care costs were increased by 42% to account for hospital overhead costs according to Dutch cost-pricing standards [21]. We pre-defined costs per CTP of €251.40 based on the following assumptions and data: €129 for the CTP acquisition [21], costs for acute care personnel (€94) [21], €20 for the CTP software license based on expert opinion. EVT costs were €9924.50 consisting of material costs and 1.5 h of personnel costs for (neuro-) interventionist, 1 anesthesiologist, 2 radiology assistants, and 2 anesthesia assistants [12, 20]. IVT costs of €950.82 were extracted from medicijnkosten.nl. Simulations started in 2022. An annual discounting rate of 4% for QALYs and 1.5% for costs was used to compute present values [22]. Inflation-based cost adjustments were made using historical and forecasted inflation rates [23, 24].

### Outcome measures

Net monetary benefit (NMB: Formula 1) at a willingness to pay (WTP) of €80,000 per QALY was the primary outcome. Secondary outcomes were the differences in cost (ΔCosts) and quality-adjusted life-years (ΔQALYs) between the intervention (NCCT + CTA + CTP-based LVO detection) and control (NCCT + CTA-based LVO detection) arm. All results were reported as cumulative values over the simulated period with median and interquartile range (IQR) per simulated patient with an LVO.

1 $$NMB=WTP*({QALY}_{NCCT+CTA+CTP}-{QALY}_{NCCT+CTA})- ({Costs}_{NCCT+CTA+CTP}-{Costs}_{NCCT+CTA})$$

### Baseline and sensitivity analyses

Mean values of the input parameters were used for the baseline simulation considering a 5-year follow-up period. A 10% increase and decrease of all input parameters were used to simulate one-way sensitivity results to assess the outcome variability due to input parameter changes. Probabilistic sensitivity analyses (PSAs) were performed to represent input parameter uncertainty in the outcome measures. For the PSAs, 1000 cohorts were sampled with replacement from the original data. All model input parameters and distributions are described in Table 1.

**Table 1:** Data sources for model input parameter estimation

Variable	Value	Distribution	Data source
CTP-based sensitivity-gain for ICA occlusions	2%/4%/6%/8%/10% 6% as baseline value	Fixed	Olive-Gadea et al [5]
CTP-based sensitivity-gain for M1 and M2 occlusions	12%/14%/16%/18%/20% 16% as baseline value	Fixed	Olive-Gadea et al [5] Bathla et al [3] Hopyan et al [4] Becks et al [1]
Number needed to image	4.3/8.3 8.3 as baseline value	Fixed	Online supplementary table S1
EVT effect	1.67 (CI: 1.21–2.3) (baseline) 1.97 (CI: 1.51–2.6) (upper) 1.37 (CI: 0.91–2.0) (lower) 2.49 (CI: 1.76–3.53)	Log-normal	Berkhemer et al [21] Goyal et al [23]
Baseline probability of stroke recurrence	Dependent on years after index ischemic stroke	Fixed	Pennlert et al [12]
HR recurrent stroke	Age and mRS dependent	Log-normal	Pennlert et al [12]
Baseline probability of death	Age, gender, and year dependent	Fixed	Dutch Royal Actuarian Society [13]
HR mortality (by mRS: 01/2/3/4/5)	1.54/2.17/3.18/4.55/6.55	Log-normal	Hong et al [14]
Inflation rate in % per year	% per year	Fixed value	Central Bureau of Statistics [17]
Costs EVT*	€9924.50	Fixed	Van den Berg. [7]
Costs IVT*	€950.82	Fixed	Van den Berg. [7]
Costs of CTP*	€251.40	Fixed	Estimated in protocol [10]
Costs year 1* (by 90 day mRS: 01/2/3/4/5/6)	33,402 (31,930)/52,804 (23,571)/82,452 (35,333)/112,414 (35,786)/96,640 (30,463)/21,112 (17,350)	Gamma	Van Voorst et al [11]
Costs year 2* (by 18 month mRS: 01/2/3/4/5/6)	5934 (15,918)/8543 (14,844)/19,235 (15,999)/43,193 (45,640)/56,425 (24,252)/423 (3196)	Gamma	Van Voorst et al [11]
Costs year 2* onward (by 18 month mRS: 01/2/3/4/5/6)	3633 (9087)/7318 (13,770)/16,276 (11,753)/31,037 (19,928)/54,997 (24,874)/374 (3118)	Gamma	Van Voorst et al [11]
QALY (by mRS: 01/2/3/4/5/6)	0.94 (0.09)/0.80 (0.17)/0.68 (0.24)/0.39 (0.26)/0.24 (0.25)/0 (0.01)	Beta	Van Voorst et al (11)

*EVT*, endovascular treatment; *HR*, hazard ratio; *IVT*, intravenous thrombolysis; *mRS*, modified Rankin Scale score; *OR*, odds ratio; *QALY*, quality-adjusted life year. *Costs are depicted for the reference year 2015. Costs per year included: in-hospital costs, outpatient clinic visits, rehabilitation, formal homecare, and costs for long term institutionalized care. Part of this table has been previously described [10, 15]

#### Dedicated PSAs

We performed dedicated PSAs to analyze the effect of major factors that affect the cost-effectiveness of CTP-based EVT-eligible occlusion detection.

A) To summarize the variations in sensitivity difference between NCCT + CTA + CTP- and NCCT + CTA-based LVO detection, we performed a systematic search on PubMed combining terms related to CTP, CTA, sensitivity, and stroke with an AND term (Supplement A). We defined ranges of sensitivity difference for ICA, M1, and M2 occlusions separately based on the literature [1–4] (Table 2). For ICA occlusions, we used a baseline sensitivity difference of 8% and varied values with increments of 2% between 4 and 12% [4]. For M1 and M2 occlusions, we used a baseline sensitivity difference of 16% and varied values with increments of 2% between 12 and 20% [2, 4].
B) We performed a sensitivity analysis for the treatment effect of EVT that was used to generate the 90-day mRS of patients that would not receive EVT in the NCCT + CTA arm (Online Supplement B). The OR for the treatment effect of EVT (OR: 1.67; 95%CI: [1.21; 2.30]) from the MR CLEAN trial [11], the trial with the most conservative EVT benefit [6], was altered with − 0.3, + 0.3, and + 0.82 [6].
C) We used the number needed to image (NNI) to accrue for the costs of all CTPs made, including CTP costs for patients not receiving EVT. We varied the NNI between 4.3 and 8.3, based on previously reported values in the literature and ambulance data from two urban regions in the Netherlands (detailed computations in Supplement C). We assumed that 50–60% of all suspected stroke patients admitted to an EVT-capable hospital have an AIS [25] (Table 3). Of all AIS patients, 24–46% have an LVO [26].

**Table 2:** Sensitivity difference between NCCT + CTA + CTP and NCCT + CTA for large vessel occlusion detection

Reference	Definition of occlusion location in study	Number of patients	Number of raters	Sensitivity-difference (%)*	Sensitivity with NCCT + CTA (%)	Sensitivity with NCCT + CTA + CTP (%)
Olive-Gadea et al [5]	ICA	22	3	8.3	91.7	100
	M1	37	3	15.6	84.4	100
Bathla et al [3]	M2 occlusion	46	2	14/18**	78/76**	91/93**
Hopyan et al[4]	ICA, ACA, M1-m4, PCA, vertebrobasilar artery, PICA, SCA, other small vessels	191	4	20	55.4	69.5
Becks et al[1]	Proximal occlusions: ICA, A1, P1, M1, M2, basilar and vertebral artery	36	3	0/6**	89/94**	89/100**

*CTA*, CT angiography; *CTP*, CT perfusion; *ACA*, anterior cerebral artery (sub-segments: A1–A3); *PCA*, posterior cerebral artery (sub-segments: P1–P4); *MCA*, middle cerebral artery (sub-segments: M1–M4); *ICA*, internal carotid artery; *PICA*, posterior inferior cerebellar artery; *SCA*, superior cerebellar artery; *AICA*, anterior inferior cerebellar artery. *Sensitivity difference was computed as the sensitivity difference between NCCT + CTA + CTP- and NCCT + CTA-based occlusion detection divided by the sensitivity of NCCT + CTA + CTP-based occlusion detection. **Results were reported for multiple raters; we reported this as the rater with the lowest and highest sensitivity-difference (lowest/highest)

**Table 3:** Proportion of acute ischemic stroke in patients suspected for stroke at an emergency ward

Acute ischemic stroke or not	Greater Rotterdam Region PRESTO [7]	Greater Amsterdam region ambulance services				
		2018	2019	2020	2021	2022 (until 19–09-2022)
Any form of acute ischemic stroke	522 (54%)	623 (50%)	712 (50%)	853 (57%)	797 (57%)	554 (60%)
Transient ischemic attack or stroke mimic	445 (46%)	634 (50%)	704 (50%)	643 (43%)	591 (43%)	369 (40%)

Data from the greater Rotterdam are extracted from the PRESTO trial. Data from the Greater Amsterdam area was obtained from a database on ambulance data and final diagnoses

## Results

### Descriptive statistics

**Fig. 2:**
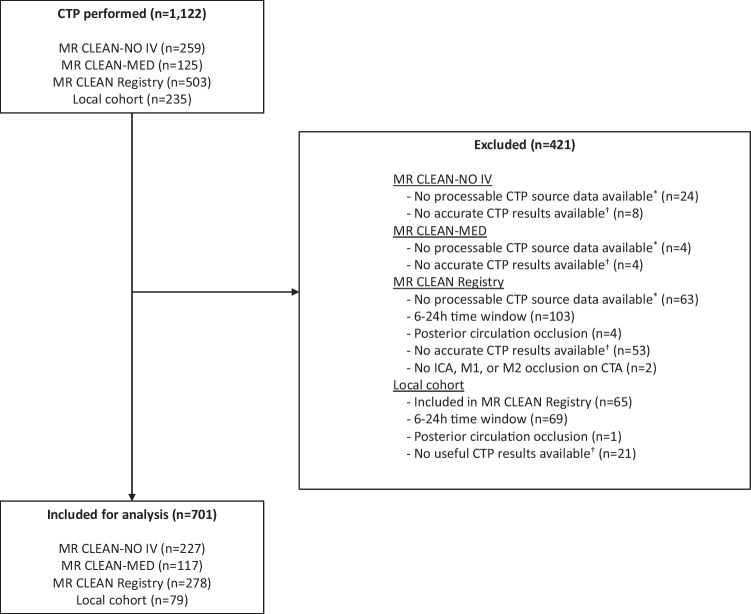
Flowchart of patient selection. ^*^CTP source data without time information or CTP source data not available due to local storage in the primary stroke center. ^†^Reasons for inaccurate CTP results include severe patient motion, severe curve truncation, no timely contrast arrival or incorrect timing CTP, or severe artifacts in CTP source data. CTP, CT perfusion; ICA, internal carotid artery

We included 701 (390/701 male, median age 72 [IQR: 62; 81]) of 1122 patients available in the CLEOPATRA database for the simulations. An inclusion flow chart is available in Fig. 2. Patients were excluded due to an onset of stroke symptoms to groin puncture time beyond 6 h (*n* = 172), absence of CTP source data (*n* = 91), no accurate CTP results after processing (*n* = 86), double inclusion (*n* = 65), and an unknown occlusion location or a posterior circulation occlusion (*n* = 7). Baseline characteristics for the total population and subgroups based on occlusion location are presented in Table 4.

**Table 4:** Descriptive statistics of the included cohort

Variable	Total population (*n* = 701)	Occlusion location			*p*-value
		ICA or ICA-T (*n* = 168)	M1 (*n* = 367)	M2 (*n* = 166)	
Age (median—IQR)	72 (62; 81)	70 (59; 77)	72 (62; 80)	73 (65; 82)	0.01
Male sex	390 (55.6%)	100 (59.9%)	200 (54.5%)	90 (54.2%)	0.44
Baseline NIHSS (median—IQR)	15 (10; 19)	17 (13; 20)	16 (10; 20)	11 (7; 17)	< 1e-5
mRS before AIS					0.53
0	222 (64.9%)	65 (73.0%)	117 (61.9%)	40 (62.5%)	
1	70 (20.5%)	16 (18.0%)	39 (20.6%)	15 (23.4%)	
2	39 (11.4%)	7 (7.9%)	25 (13.2%)	7 (10.9%)	
3	11 (3.2%)	1 (1.1%)	8 (4.2%)	2 (3.1%)	
Baseline ASPECTS (median-IQR)	9 (8; 10)	8 (7; 10)	9 (8; 10)	10 (9; 10)	< 1e-5
Baseline collateral score					0.02
0	21 (6.2%)	10 (11.4%)	11 (5.9%)	0 (0%)	
1	90 (26.6%)	23 (26.1%)	55 (29.6%)	12 (18.8%)	
2	160 (47.3%)	40 (45.5%)	88 (47.3%)	32 (50.0%)	
3	67 (19.8%)	15 (17.0%)	32 (17.2%)	20 (31.2%)	
Ischemic core volume in mL (mean—SD)	13 (5; 33)	18 (6; 55)	11 (5; 31)	13 (5; 27)	0.01
Penumbra volume in mL (mean—SD)	113 (128)	145 (247)	112 (45)	82 (42)	< 1e-3
Core-penumbra mismatch ratio (median—IQR)	9 (4; 19)	7 (4; 19)	10 (5; 21)	7 (4; 15)	< 0.01
Onset to groin puncture time in minutes (median—IQR)	158 (115; 230)	157 (120; 226)	153 (114; 228)	173 (122; 238)	0.45
Door to groin puncture time in minutes (mean—SD)	67 (50; 90)	68 (51; 96)	64 (49; 86)	70 (53; 104)	0.11
Duration of procedure in minutes (median—IQR)	48 (31; 71)	60 (40; 88)	42 (29; 65)	45 (31; 66)	< 1e-3
Intravenous thrombolysis administration	382 (67.3%)	86 (66.2%)	198 (66.7%)	98 (69.5%)	0.80
Modified Rankin Scale 90-days after EVT number (%)					0.45
0	47 (7.3%)	7 (4.6%)	28 (8.3%)	12 (7.8%)	
1	115 (17.8%)	21 (13.8%)	61 (18.0%)	33 (21.4%)	
2	154 (23.9%)	35 (23.0%)	87 (25.7%)	32 (20.8%)	
3	69 (10.7%)	20 (13.2%)	35 (10.3%)	14 (9.1%)	
4	76 (11.8%)	20 (13.2%)	43 (12.7%)	13 (8.4%)	
5	42 (6.5%)	11 (7.2%)	20 (5.9%)	11 (7.1%)	
6	142 (22.0%)	38 (25.0%)	65 (19.2%)	39 (25.3%)	

*AIS*, acute ischemic stroke; *ASPECTS*, Alberta Stroke Program Early CT Score; *IQR*, interquartile range; *mRS*, modified Rankin Scale; *NIHSS*, National Institute of Health Stroke; *SD*, standard deviation; *EVT*, endovascular treatment

### Baseline model and one-way sensitivity

**Fig. 3:**
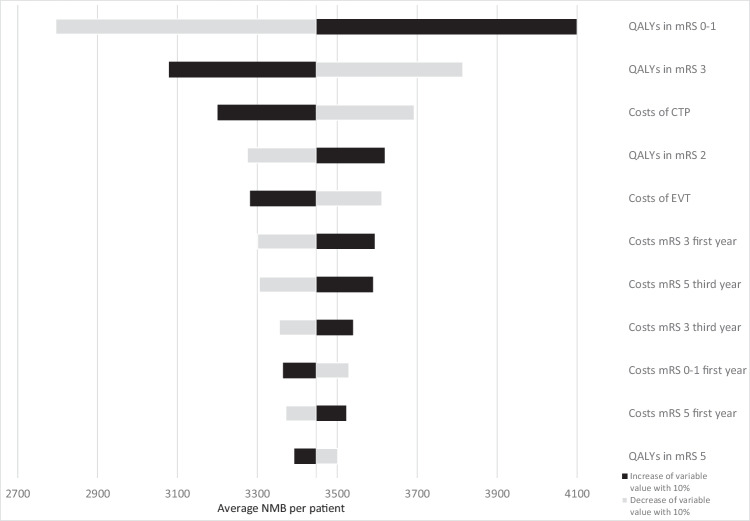
Tornado diagram of the one-way sensitivity analyses. Changes in average NMB compared to the baseline (€3447) are depicted for a 10% increase (black) and decrease (gray) of the ten most influential model input variables. A 5-year horizon was used with an NNI of 8.3 and a baseline sensitivity gain. Variations in NNI, sensitivity gain, and EVT effect were not considered for this analysis. EVT, endovascular treatment; QALY, quality-adjusted life-years; mRS, modified Rankin Scale; CTP, CT perfusion; NMB, net monetary benefit

Using the mean input values, an NNI of 8.3, and 5 years of follow-up for the baseline simulations resulted in a gain of health (ΔQALY: 0.049) and higher costs (ΔCosts: €482), with a positive NMB (€3447) when NCCT + CTA + CTP would be used compared to NCCT + CTA for LVO detection. Figure 3 describes the ten most influential model parameters in a one-way sensitivity analysis. The amount of QALYs attributed to mRS 0–3 were the most important factors affecting the NMB. Costs of CTP and EVT were the most influential cost factors affecting the NMB; long-term follow-up care costs only had a limited effect on the NMB. Figure S3 (Online Supplement G) contains a Kaplan-Maier plot describing the simulated 10-year survival.

### Probabilistic sensitivity analysis

**Fig. 4:**
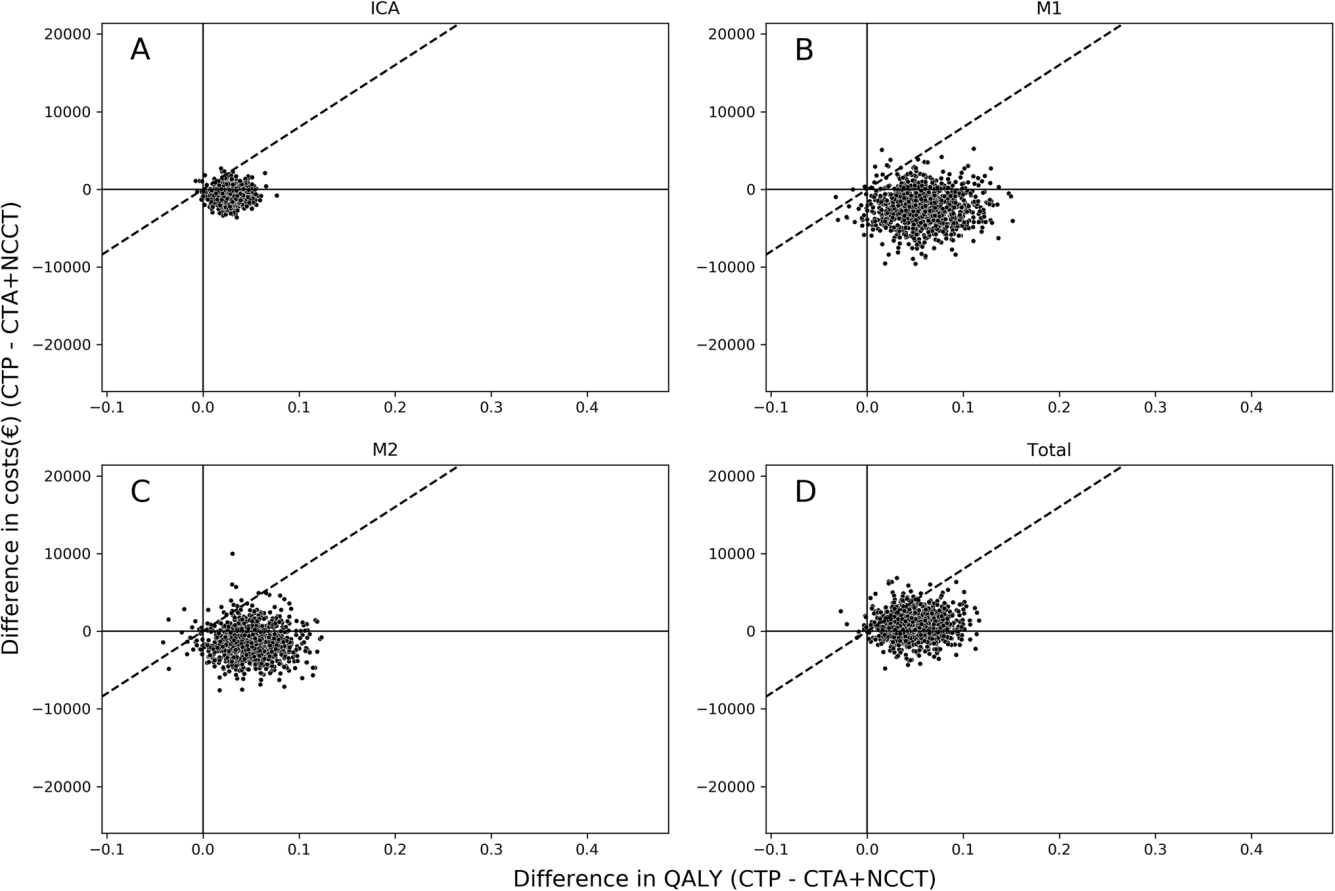
Incremental cost-effectiveness ratio (ICER) plot per occlusion location. Incremental cost-effectiveness ratio plots are presented for (**A**) ICA, (**B**) M1, (**C**) M2, and (**D**) all simulated patients together. Simulations considered the baseline sensitivity gain per occlusion location, and a 5-year follow-up period. Panels **A–C** do not include the CTP screening costs; panel **D** does include the CTP screening costs using the NNI multiplier (NNI = 8.3). Positive values represent more costs or QALYs when CTP is included in an imaging protocol consisting of NCCT and CTA for occlusion detection. The dashed diagonal line represents the willingness to pay of €80,000 per QALY

The incremental cost-effectiveness ratio plots per occlusion location and for all occlusion locations together are visualized in Fig. 4. Considering an NNI of 8.3, the baseline sensitivity difference, and a follow-up horizon of 5 years resulted per EVT-eligible LVO patient in an increase in costs (ΔCosts median: €777, IQR: [€ − 290; 1825]), a health gain (ΔQALY median: 0.048, IQR: [0.032; 0.064]), and a positive NMB (median: €3015, IQR: [€1455; €4779) when NCCT + CTA + CTP would be used compared to NCCT + CTA for LVO detection. Using similar settings but a 10-year follow-up horizon resulted in a cost-saving (ΔCosts median: € − 2671, IQR: [€ − 4721; € − 731]), a health gain (ΔQALY median: 0.073, IQR: [0.044; 0.104]), and a positive NMB (median: €8436, IQR: [€5565; €11,876]).

### Dedicated sensitivity analyses

**Fig. 5:**
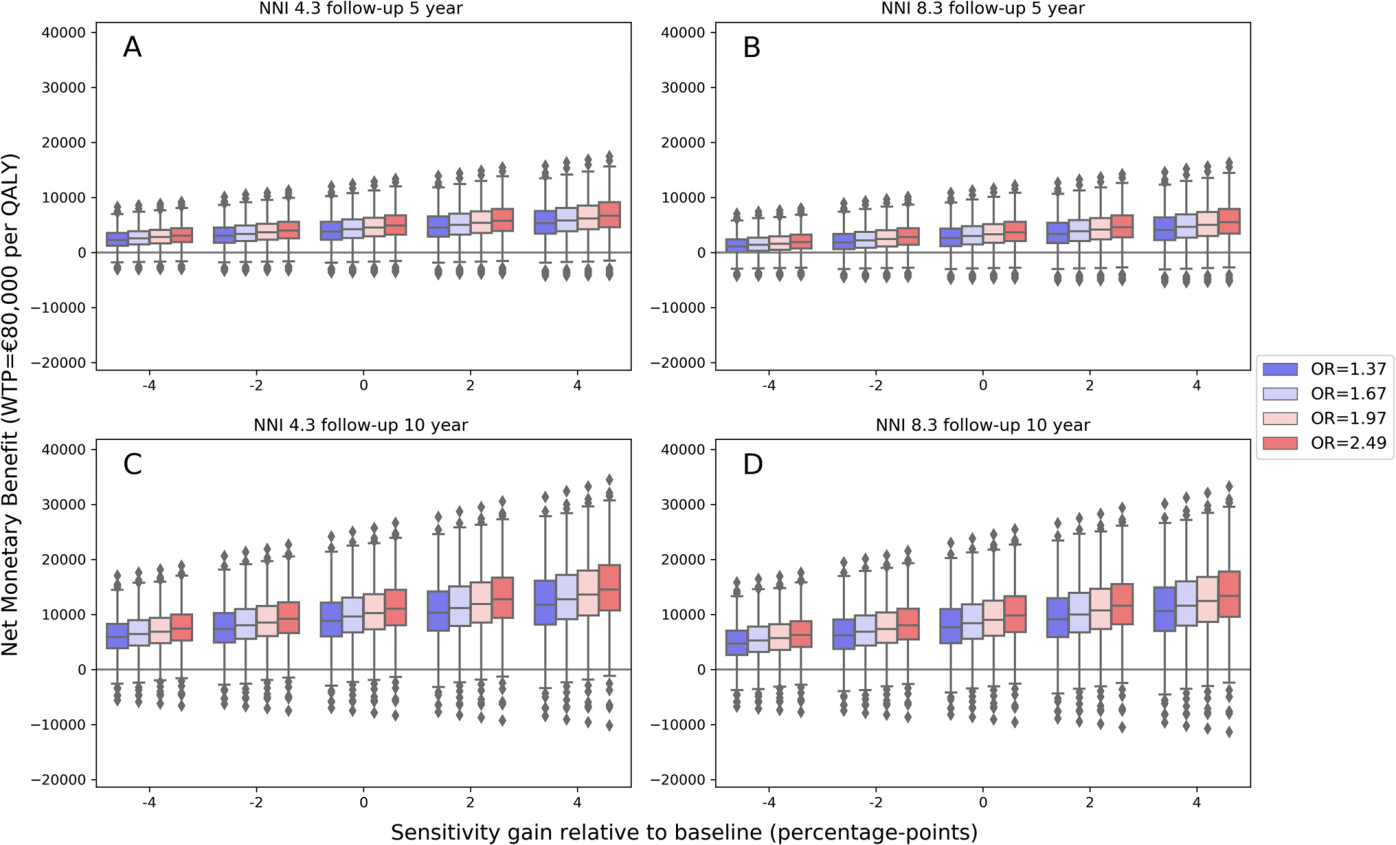
Net monetary benefit for dedicated probabilistic sensitivity analyses. Each panel shows the NMB at a willingness to pay of €80,000 per quality-adjusted life years (QALY) on the y-axis. A positive net monetary benefit implies that the additional costs of CTP-based screening, EVT, and long-term care costs are lower than the health gain. On the x-axis, the percentage point in sensitivity difference relative to the baseline values of additional CTP (NCCT + CTA + CTP) compared to NCCT + CTA is depicted. The baseline sensitivity difference was 6% for ICA occlusions and 16% for M1 and M2 occlusions. The colors represent the median OR for the treatment effect used for simulations. Panels **A–D** depict varying NNI and years of follow-up. (**A**) NNI of 4.3 considering 5-year follow-up. (**B**) NNI of 8.3 considering 5-year follow-up. (**C**) NNI of 4.3 considering 10-year follow-up. (**D**) NNI of 8.3 considering 10-year follow-up. NNI, number of patients needed to image; OR, odds ratio; WTP, willingness to pay; QALY, quality-adjusted life years; NMB, net monetary benefit

Variations in NMB due to the follow-up horizon, NNI, and sensitivity difference are graphically presented in Fig. 5. Table 5 contain the results for ΔQALY, ΔCosts, NMB, and the fraction of the simulations that were cost-effective (below the WTP line; an NMB > 0) at a WTP of €80,000 for varying scenarios. More extensive results for varying model parameters are described in Online Supplement G.

**Table 5:** Probabilistic sensitivity results for different scenarios

Model settings			Simulation results			
Years of follow-up	NNI	Sensitivity gain relative to baseline (%-points)	ΔCosts in € median (IQR)	ΔQALY median (IQR)	NMB in € median (IQR)	Fraction of simulations cost-effective (cost-effective/1000)
10	4.3	+ 8	− 6861 (− 10,016; − 3883)	0.115 (0.07; 0.163)	15,973 (11,517; 21,332)	0.992
10	4.3	+ 4	− 5356 (− 7972; − 2899)	0.094 (0.057; 0.134)	12,771 (9178; 17,186)	0.991
10	4.3	0	− 3857 (− 5907; − 1916)	0.073 (0.044; 0.104)	9621 (6750; 13,061)	0.987
10	4.3	− 4	− 2344 (− 3807; − 960)	0.052 (0.031; 0.075)	6451 (4384; 8974)	0.98
10	4.3	− 8	− 859 (− 1733; − 15)	0.031 (0.018; 0.045)	3289 (2006; 4819)	0.956
10	8.3	+ 8	− 5676 (− 8830; − 2698)	0.115 (0.07; 0.163)	14,787 (10,331; 20,147)	0.984
10	8.3	+ 4	− 4171 (− 6786; − 1713)	0.094 (0.057; 0.134)	11,585 (7992; 16,001)	0.981
10	8.3	0	− 2671 (− 4721; − 731)	0.073 (0.044; 0.104)	8436 (5565; 11,876)	0.973
10	8.3	− 4	− 1158 (− 2621; 225)	0.052 (0.031; 0.075)	5266 (3199; 7789)	0.953
10	8.3	− 8	325 (− 548; 1169)	0.031 (0.018; 0.045)	2103 (821; 3634)	0.864
5	4.3	+ 8	− 1515 (− 3130; 113)	0.075 (0.051; 0.099)	7451 (5051; 10,223)	0.98
5	4.3	+ 4	− 965 (− 2314; 357)	0.062 (0.042; 0.081)	5839 (3863; 8106)	0.977
5	4.3	0	− 407 (− 1476; 640)	0.048 (0.032; 0.064)	4200 (2640; 5965)	0.969
5	4.3	− 4	126 (− 639; 888)	0.034 (0.023; 0.046)	2569 (1450; 3853)	0.934
5	4.3	− 8	686 (209; 1152)	0.021 (0.014; 0.028)	935 (260; 1752)	0.817
5	8.3	+ 8	− 329 (− 1945; 1298)	0.075 (0.051; 0.099)	6265 (3865; 9038)	0.963
5	8.3	+ 4	220 (− 1128; 1543)	0.062 (0.042; 0.081)	4654 (2678; 6921)	0.946
5	8.3	0	777 (− 290; 1825)	0.048 (0.032; 0.064)	3015 (1455; 4779)	0.901
5	8.3	− 4	1312 (545; 2074)	0.034 (0.023; 0.046)	1384 (265; 2667)	0.801
5	8.3	− 8	1872 (1394; 2337)	0.021 (0.014; 0.028)	− 249 (− 925; 566)	0.416

From top to bottom, the scenarios become less favorable for additional CTP compared to CTA- and NCCT-based endovascular treatment-eligible occlusion detection. All scenarios considered an adjusted common OR of 1.67 for the treatment effect of EVT [21]. An extended version of this table is available in Online supplement G. *NNI*, number needed to image before one endovascular treatment eligible occlusion is detected. The sensitivity gain of additional CTP represents the percentage-point difference between baseline values of 8% for ICA and 16% for M1 and M2 occlusions. *NMB*, net monetary benefit at a willingness to pay of €80,000 per QALY; *Δ*, the difference between additional CTP- and CTA + NCCT-based occlusion detection. *ΔCosts*, difference in costs; negative values imply a cost saving if CTP is added; *ΔQALYs*, difference in quality-adjusted life years; negative value implies a gain of health if CTP is added

Between the upper and lower NNI bound additional costs or savings differed (ΔCosts 10-year follow-up: NNI = 4.3; median: € − 3857, IQR: [€ − 5907; € − 1916] vs. NNI = 8.3; median: € − 2671, IQR: [€ − 4721; € − 731]) while the QALYs were the same (ΔQALY median: 0.073, IQR: [0.044; 0.104]). Variations in sensitivity difference resulted in different health gains (ΔQALYs 10-year follow-up: sensitivity difference = baseline median: 0.073, IQR: [0.044; 0.104] vs. sensitivity difference = (baseline − 4%) median: 0.052, IQR: [0.031; 0.075] vs. sensitivity difference = (baseline + 4%) median: 0.094, IQR: [0.057–0.134]). Furthermore, variations in EVT-effect relative to the baseline effect found in the MR CLEAN trial (baseline OR: 1.67; 95%CI: [1.21; 2.30]) resulted in a limited difference in cost savings (NNI = 8.3 10-year follow-up: ΔCosts EVT-effect baseline median: € − 2671, IQR: [€ − 4721; € − 731]; baseline − 0.3 median: € − 2683, IQR: [€ − 4715; € − 836]; baseline + 0.3 median: € − 2592, IQR: [€ − 4622; € − 657]) and more profound variations in health gains (10-year follow-up: ΔQALYs baseline median: 0.073, IQR: [0.044; 0.104]; baseline − 0.3 median: 0.062, IQR: [0.034; 0.094]; baseline + 0.3: 0.082, IQR [0.052; 0.113]). NCCT + CTA + CTP-based LVO detection would not be cost-effective if we considered 5-year follow-up, an NNI of 8.3, and a sensitivity gain 8% below the baseline (ΔCosts median: €1872, IQR: [€1394; €2337]; ΔQALYs median: 0.021, IQR: [0.014; 0.028]; NMB median: € − 249, IQR: [€ − 925; €566]).

## Discussion

In this cohort study with model-based health economic evaluation, we found that adding CTP to an imaging regime of CTA and NCCT for EVT-eligible large vessel occlusion detection followed by endovascular treatment, resulted in a cost savings (ΔCosts median: € − 2671, IQR: [€ − 4721; € − 731]), a health gain (ΔQALY median: 0.073, IQR: [0.044; 0.104]), and a positive net monetary benefit (median: €8436, IQR: [5565; 11,876]) during a 10-year follow-up horizon considering a healthcare payer perspective per patient with an LVO. Costs and health effects per CTP screened patients were small but unevenly distributed as only the group of patients with a missed occlusion would benefit. Around 2350 patients receive EVT per year in the Netherlands [27], for each year that CTP would be used to detect LVOs eligible for EVT 172 QALYs (IQR: [103; 244]) and €9.1 million (IQR: €13.9mln; €4.5mln) could be saved.

Similar to previous research, the use of CTP was found cost-effective [9, 10]. However, this study provides a more detailed description of factors causing changes in costs and health effects. To interpret these results, we need to consider the currently available evidence. First, NNI estimates might differ; a higher ischemic stroke prevalence or less CTPs with uninterpretable results would result in a lower NNI and thus result less CTPs per detected LVO and lower costs. Second, the benefit in LVO detection sensitivity of additional CTP to a diagnostic workup consisting of NCCT and CTA for AIS screening from previous reader studies varied depending on the physician’s experience [1–4]. The value of CTP-based screening might be higher for physicians with less experience in neuroimaging assessments. In this study we reported a wide variety of sensitivity differences as the current evidence comparing NCCT + CTA + CTP- with NCCT + CTA-based LVO detection is limited by a small sample size and suboptimal research designs [1–4]. Third, we used conservative ORs for the EVT treatment effect based on the MR CLEAN trial [12]. In recent years, EVT workflows have improved, improving the treatment effect of EVT [28]. By underestimating the effect of EVT, we might allow for better generalizability of our findings to settings with different stroke populations or physician’s experience with EVT, such as primary stroke centers or stroke centers in developing countries. Namely, due to the transfer of patients, the onset to groin time increases, negatively affecting the EVT effect [29]. Fourth, the variety of occlusion subtypes in our population might be different from the occlusion subtype distribution in other populations [6]. Fifth, costs and health effects due to negative side effects related to CTP were not considered. Allergic reactions and renal insufficiency due to a higher dose of intravenously administered contrast medium may be harmful [30, 31]. Furthermore, additional radiation exposure due to CTP might cause harm when considering long-term follow-up [32]. Since these side effects occur in a very small portion of the population, often have limited long-term effects, or occur in the far future, we expect this to only have a minor effect on the costs and health effects in this study. Sixth, current trials studying EVT for more distal occlusions might result in even higher health gains due to CTP-based occlusion detection [33, 34].

Our study has limitations. This is a model-based study using an observational cohort of only patients receiving EVT in the Netherlands, our findings might deviate in prospectively based trial cost-effectiveness analyses and in other patient populations. Specifically, we did not have data on long-term functional outcome follow-up, micro-level-estimated costs, nor did we include patients that did not receive EVT due to a missed LVO. Furthermore, we used cost data from a healthcare payer perspective neglecting indirect costs. Indirect costs related to poor patient outcomes such as a reduction in labor participation and higher out-of-pocket healthcare expenses might result in an even higher benefit of CTP-based EVT-eligible LVO screening.

Future research should analyze alternative diagnostic imaging strategies compared to the direct use of NCCT, CTA, and CTP used in this study. CTP could be considered after an inconclusive CTA and NCCT examination or in combination with patient characteristics. Although this study focused on radiologist-based CTP examination, recently developed artificial intelligence–based software might also improve occlusion detection and could therefore be cost-effective. Comparative studies are required to define the optimal occlusion detection strategy. Since we only considered EVT-eligible LVOs, future studies should consider the value of CTP for detecting EVT-eligible distal vessel occlusions or posterior circulation occlusions — which are generally more difficult to detect. Finally, future work should aim to determine stroke subtype statistics to compute the NNI and assess determinants for varying sensitivity differences due to CTP in a large reader study.

## Conclusion

In this model-based health economic evaluation study, detecting more EVT eligible LVOs using CTP-based screening for patients presenting within 6 h after symptom onset was cost-effective due to a health gain and limited additional costs to a cost saving over 10-year follow-up. Health gains and cost savings might be more favorable for a lower number needed to image, a longer follow-up horizon, and a higher sensitivity difference of additional CTP compared to NCCT and CTA based large vessel occlusion detection.

### Supplementary Information

Below is the link to the electronic supplementary material.

Supplementary file1 (PDF 682 KB)

## Abbreviations

AIS: Acute ischemic stroke
CLEOPATRA: Cost-effectiveness of CT perfusion for Patients with Acute Ischemic Stroke
CTA: CT angiography
CTP: CT perfusion
EVT: Endovascular treatment
ICA: Internal carotid artery
ICA-T: Terminus of ICA
ICER: Incremental cost-effectiveness ratio
IQR: Interquartile range
LVO: Large vessel occlusion
mRS: Modified Rankin Scale
NCCT: Non-contrast CT
NMB: Net monetary benefit
NNI: Number needed to image
PSA: Probabilistic sensitivity analysis
QALY: Quality-adjusted life year
ΔCosts: Differences in cost
ΔQALYs: Differences in quality-adjusted life years

## Declarations

### Guarantor

The scientific guarantor for this study is Charles BLM Majoie.

### Statistics and biometry

One of the authors has significant statistical expertise: Erik Buskens, University Medical Center Groningen, Department of Epidemiology, coauthor of this manuscript.

### Study subjects or cohorts overlap

Of all 701 patients considered in this study, data from 622 have previously been reported. Patients were included in the MR CLEAN NO IV trial (*n* = 227), MR CLEAN MED trial (*n* = 117), and MR CLEAN Registry (*n* = 278). All included patients were selected as a subset of the overall MR CLEAN NO IV, MR CLEAN MED, and MR CLEAN Registry populations. We used patients included in these studies to extract population statistics (age, sex) and functional outcomes that, combined with literature, were used to simulate long-term functional outcome, costs, and QALYs. Based on simulations we describe the effect on costs and QALYs of various scenarios for screening patients with CTP. Current other studies did not have related research aims or hypotheses. We selected these patients based on the presence of CTP before EVT to describe a realistic cohort of patients that receive CTP. Patients included in the MR CLEAN NO IV (*n* = 227) and MR CLEAN MED (*n* = 117) have been studied in a randomized controlled trial setting for the effect of intravenous alteplase (0.9 mg/kg) before EVT and the effect of heparin and aspirin before endovascular treatment respectively. Functional outcome and population characteristics of the patients included MR CLEAN Registry (*n* = 278) have been reported before. A similar cost-effectiveness analysis for CTP based on the same population is under review. However, this other study considers CTP for EVT patient selection and not for EVT eligible occlusion detection.

There exists a pre-print, 10.1101/2023.03.16.23287253.

### Methodology

- Retrospective
- Multicenter study

## References

[1] Becks MJ, Manniesing R, Vister J (2019). Brain CT perfusion improves intracranial vessel occlusion detection on CT angiography. J Neuroradiol.

[2] Bathla G, PillenahalliMaheshwarappa R, Soni N (2022). Do CT perfusion maps increase accuracy for detection of M2-MCA occlusions in acute ischemic stroke?. J Stroke Cerebrovasc Dis.

[3] Hopyan J, Ciarallo A, Dowlatshahi D (2010). Certainty of stroke diagnosis: incremental benefit with CT perfusion over noncontrast CT and CT angiography. Radiology.

[4] Olive-Gadea M, Requena M, Diaz F (2021). Systematic CT perfusion acquisition in acute stroke increases vascular occlusion detection and thrombectomy rates. J Neurointerv Surg.

[5] Powers WJ, Rabinstein AA, Ackerson T (2018). Guidelines for the early management of patients with acute ischemic stroke: a guideline for healthcare professionals from the American Heart Association/American Stroke Association. Stroke.

[6] Goyal M, Menon BK, van Zwam WH (2016). Endovascular thrombectomy after large-vessel ischaemic stroke: a meta-analysis of individual patient data from five randomised trials. Lancet.

[7] Ospel JM, Kunz WG, McDonough RV (2023). Cost-effectiveness of endovascular treatment in large vessel occlusion stroke with mild prestroke disability: results from the HERMES Collaboration. Stroke.

[8] Van Den Berg LA, Berkhemer OA, Fransen PSS (2022). Economic evaluation of endovascular treatment for acute ischemic stroke. Stroke.

[9] Boltyenkov AT, Martinez G, Pandya A (2021). Cost-consequence analysis of advanced imaging in acute ischemic stroke care. Front Neurol.

[10] Martinez G, Katz JM, Pandya A (2021). Cost-effectiveness study of initial imaging selection in acute ischemic stroke care. J Am Coll Radiol.

[11] Berkhemer OA, Fransen PSS, Beumer D (2015). A randomized trial of intraarterial treatment for acute ischemic stroke. N Engl J Med.

[12] Koopman MS, Hoving JW, van Voorst H et al (2022) Cost-effectiveness of CT perfusion for patients with acute ischemic stroke (CLEOPATRA)-Study protocol for a healthcare evaluation study. Eur Stroke J 10.1177/23969873221092535 PMC9134782 35647320

[13] LeCouffe NE, Kappelhof M, Treurniet KM (2021). A randomized trial of intravenous alteplase before endovascular treatment for stroke. N Engl J Med.

[14] van der Steen W, van der Sluijs PM, van de Graaf RA (2022). Safety and efficacy of periprocedural antithrombotics in patients with successful reperfusion after endovascular stroke treatment. J Stroke Cerebrovasc Dis.

[15] Jansen IGH, Mulder MJHL, Goldhoorn RJB (2018) Endovascular treatment for acute ischaemic stroke in routine clinical practice: prospective, observational cohort study (MR CLEAN Registry). BMJ 360:10.1136/bmj.k949 PMC5844245 29523557

[16] Van Voorst H, Kunz WG, Van Den Berg LA et al (2020) Quantified health and cost effects of faster endovascular treatment for large vessel ischemic stroke patients in the Netherlands. J Neurointerv Surg 1–8 10.1136/neurintsurg-2020-017017 PMC8606465 33479037

[17] Pennlert J, Eriksson M, Carlberg B, Wiklund PG (2014). Long-term risk and predictors of recurrent stroke beyond the acute phase. Stroke.

[18] Genootschap KA, Prognosetafel AG (2018). www ag-ai nl [Internet]. [accessed 2020 Sep 1]. 2018; Available from: https://www.ag-ai.nl/view.php?action=view&Pagina_Id=888

[19] Hong KS, Saver JL (2010). Years of disability-adjusted life gained as a result of thrombolytic therapy for acute ischemic stroke. Stroke.

[20] Van Voorst H, Kunz WG, Van Den Berg LA (2021). Quantified health and cost effects of faster endovascular treatment for large vessel ischemic stroke patients in the Netherlands. J Neurointerv Surg.

[21] Roijen LH, Van der Linden N (2016). Richtlijn voor het uitvoeren van economische evaluaties in de gezondheidszorg

[22] Hakkaart-van Roijen L, van der Linden N, Bouwmans C, Kanters T, Swan Tan S (2016) Kostenhandleiding: Methodologie van kostenonderzoek en referentieprijzen voor economische evaluaties in de gezondheidszorg. Dutch Natl Health Care Inst

[23] (2020) Dutch Central Bureau of Statistics. Consumer price index [Internet]. [cited 2020 Sep 1]. Available from: opendata.cbs.nl/statline

[24] Zorguitgaven | Volksgezondheid Toekomst Verkenning [Internet]. [cited 2020 Apr 1]. Available from: https://www.vtv2018.nl/zorguitgaven

[25] Duvekot MHC, Venema E, Rozeman AD (2021). Comparison of eight prehospital stroke scales to detect intracranial large-vessel occlusion in suspected stroke (PRESTO): a prospective observational study. Lancet Neurol.

[26] Rennert RC, Wali AR, Steinberg JA (2019). Epidemiology, natural history, and clinical presentation of large vessel ischemic stroke. Clin Neurosurg.

[27] (2021) Jaarcijfers acuut herseninfarct [Internet]. Hart en Vaat cijfers. [accessed 2023 May 16]. Available from: https://www.hartenvaatcijfers.nl/jaarcijfers/jaarcijfers-acuut-herseninfarct-f323d

[28] Janssen PM, Venema E, Di Dippel WJ (2019). Effect of workflow improvements in endovascular stroke treatment: a systematic review and meta-analysis. Stroke.

[29] Saver JL, Goyal M, Van Der Lugt A (2016). Time to treatment with endovascular thrombectomy and outcomes from ischemic stroke: ameta-analysis. JAMA.

[30] Hopyan JJ, Gladstone DJ, Mallia G (2008). Renal safety of CT angiography and perfusion imaging in the emergency evaluation of acute stroke. AJNR Am J Neuroradiol.

[31] Mortelé KJ, Oliva MR, Ondategui S, Ros PR, Silverman SG (2005). Universal use of nonionic iodinated contrast medium for CT: evaluation of safety in a large urban teaching hospital. AJR Am J Roentgenol.

[32] Sowby FD (1981). Annals of the ICRP. Ann ICRP.

[33] (2022) EndovaSCular TreAtment to imProve outcomEs for Medium Vessel Occlusions (ESCAPE-MeVO Trial) - Full Text View - ClinicalTrials.gov [Internet]. [accessed 2022 Jul 22]. Available from: https://clinicaltrials.gov/ct2/show/NCT05151172

[34] (2022) DISTAL: A landmark study looking to elucidate endovascular treatment outcomes in MeVO stroke [Internet]. [accessed 2022 Jul 22]. Available from: https://neuronewsinternational.com/distal-study-evt-mevo-stroke/

